# Identification of lactate metabolism-related subtypes and development of a lactate-related prognostic indicator of lung adenocarcinoma

**DOI:** 10.3389/fgene.2022.949310

**Published:** 2022-08-24

**Authors:** Xiaoyan Chang, Tong Lu, Ran Xu, Chenghao Wang, Jiaying Zhao, Linyou Zhang

**Affiliations:** Department of Thoracic Surgery, The Second Affiliated Hospital of Harbin Medical University, Harbin, China

**Keywords:** immune-related genes, immune checkpoint blockade therapy, lung adenocarcinoma, prognosis biomarker, immune cell infiltration

## Abstract

**Background:** Increasing evidence supports that lactate plays an important role in tumor proliferation, invasion and within the tumor microenvironment (TME). This is particularly relevant in lung adenocarcinoma (LUAD). Therefore, there is a current need to investigate lactate metabolism in LUAD patients and how lactate metabolism is affected by different therapies.

**Methods:** Data from LUAD patients were collected from The Cancer Genome Atlas (TCGA) and patients were divided into two subtypes according to 12 lactate metabolism-related genes to explore the effect of lactate metabolism in LUAD. We established a lactate-related prognostic indicator (LRPI) based on different gene expression profiles. Subsequently, we investigated associations between this LRPI and patient survival, molecular characteristics and response to therapy. Some analyses were conducted using the Genomics of Drug Sensitivity in Cancer (GDSC) database.

**Results:** The two LUAD subtypes exhibited different levels of lactate metabolism, in which patients that displayed high lactate metabolism also had a worse prognosis and a poorer immune environment. Indeed, LRPI was shown to accurately predict the prognosis of LUAD patients. Patients with a high LRPI showed a poor prognosis coupled with high sensitivity to chemotherapy using GDSC data. Meanwhile, these patients exhibited a high responsiveness to immunotherapy in TMB (Tumor mutation burden) and TIDE (Tumor Immune Dysfunction and Exclusion) analyses.

**Conclusion:** We validated the effect of lactate metabolism on the prognosis of LUAD patients and established a promising biomarker. LRPI can predict LUAD patient survival, molecular characteristics and response to therapy, which can aid the individualized treatment of LUAD patients.

## Introduction

Lung cancer is the leading cause of cancer-related deaths in the United states, with an estimated 609,360 deaths from 2019 to 2022, and 350 deaths per day (Siegel et al., 2022). Histologically, lung cancer is often divided into two types: small cell lung cancer (SCLC) and non-small cell lung cancer (NSCLC). Lung adenocarcinoma (LUAD) is the most common subtype of NSCLC, displaying a high tumor heterogeneity, which poses a great obstacle to the elucidation of its oncogenic mechanisms (Calvayrac et al., 2017). Before the wide-scale clinical application of immunotherapy, the treatment methods available for LUAD patients included surgical resection, chemotherapy, radiotherapy and targeted therapy, with very limited efficacy (Ha et al., 2016; Hellmann et al., 2017; Chen et al., 2020). Nevertheless, the surge of novel immune checkpoint inhibitors and targeted therapy has improved the survival of LUAD patients (Herbst et al., 2018). Given the heterogeneous biology of tumor cells and the tumor microenvironment (TME) of LUAD, the response of different patients to various treatments is also variable (Marusyk et al., 2020). Therefore, it is necessary to segment this disease into different subtypes and build models to predict patient prognosis and treatment response (Tang et al., 2017).

Lactate was long regarded as an end product of cellular glycolysis. Otto Warburg was the first to propose lactate as a cancer biomarker and coined the term Warburg metabolism, thus elucidating how tumor cells produce lactate. Recent evidence suggests that lactate broadly affects different biological processes during tumor development (Ippolito et al., 2019). Lactate promotes the proliferation and invasion of LUAD by promoting the metabolic activity of tumor cells (Chen et al., 2016; Morandi et al., 2016), driving tumor drug resistance (Apicella et al., 2018), and inhibiting the cytolytic activity of immune cells (Crane et al., 2014; Brand et al., 2016). Therefore, the stratification of LUAD patients according to lactate-related genes could predict survival outcomes and guide treatment.

In this study, we constructed a lactate-related gene prognostic indicator (LRPI) for LUAD patients to predict patient prognosis, molecular characteristics, and response to treatment. We screened genes related to prognosis that were also related to lactate metabolism to classify LUAD patients into two subtypes. Next, we assessed the association between different patient subtypes and survival to construct the LRPI. We subsequently conducted an extensive study of the stratified survival characteristics of LUAD patients, analyzed patient mutational spectrum and predicted driver mutation genes to assess patient response to multiple treatments. Altogether, we show that LRPI is a good prognostic tool for LUAD patients and that it might be helpful to guide treatment.

## Materials and methods

### Data acquisition

**FIGURE 1:**
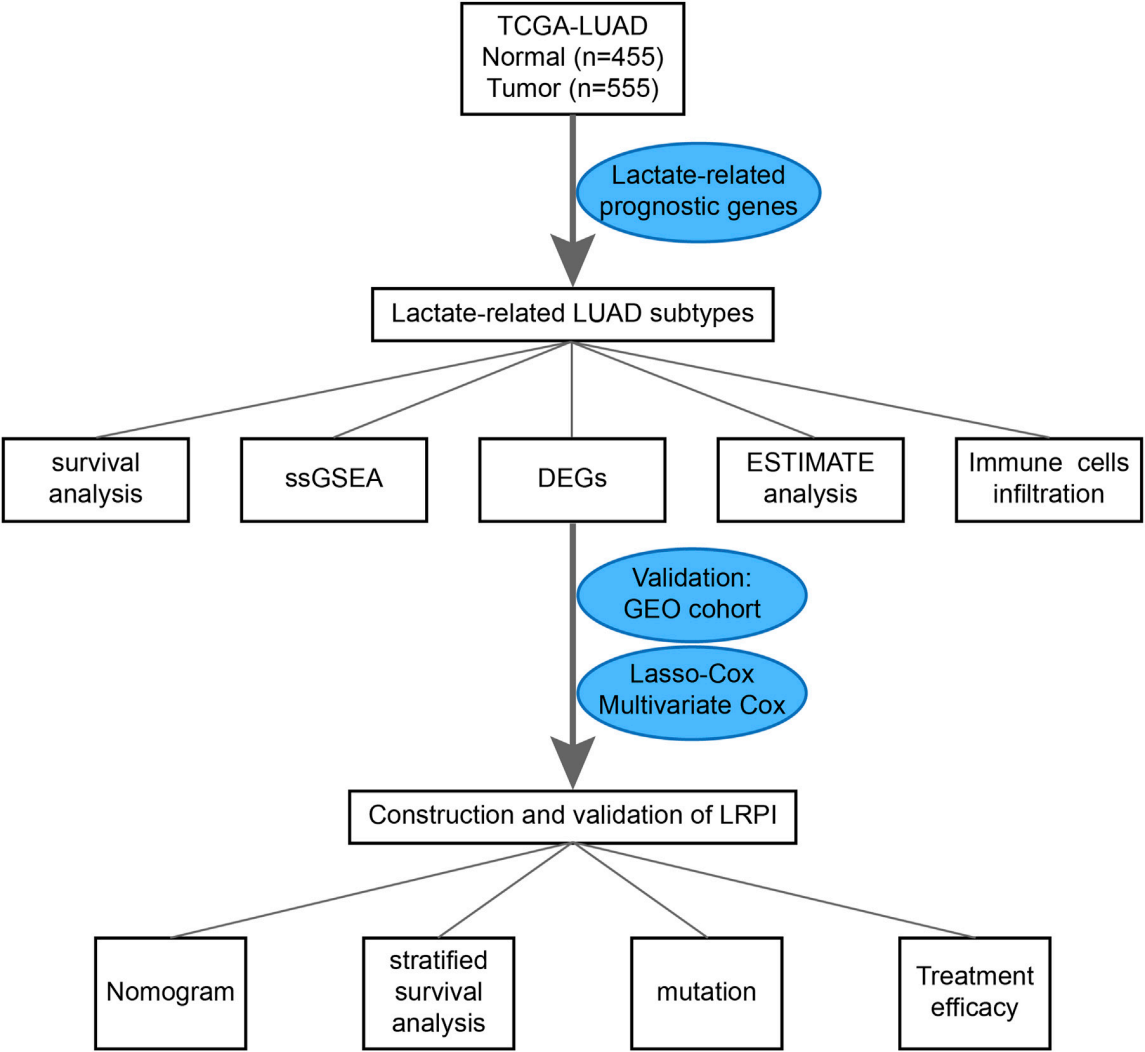
The flowchart showed the design of the study.

RNA-seq data from LUAD and normal lung tissues, clinical data, LUAD simple nucleotide variation (“Masked Somatic Mutation” data preprocessed by VarScan2), and “Masked Copy Number Segment” data were downloaded from the TCGA GDC database (https://portal.gdc.cancer.gov/). Moreover, data were also downloaded from the Gene Expression Omnibus (GEO) cohort (GSE72094, https://www.ncbi.nlm.nih.gov/geo/). The list of lactate-related genes was extracted from the GOBP LACTATE METABOLIC PROCESS and HP INCREASED SERUM LACTATE gene sets, both of which were downloaded from the MsigDB database (http://www.gsea-msigdb.org). A flowchart for this design is presented in Figure 1.

### Identification of lactate-related subtypes in LUAD samples

First, we constructed an expression matrix of lactate-related genes for all samples within the TCGA dataset. To screen genes associated with the prognosis of LUAD patients, we performed an univariate Cox regression on the obtained expression matrix to identify genes that were correlated with patient prognosis. We performed a consensus clustering of all tumor patients in the TCGA cohort based on the expression matrix of lactate genes related to prognosis. ConsensusClusterPlus function of ConsensusClusterPlus package of R software (Wilkerson and Hayes, 2010) is used for consensus clustering and the important parameters are set as follows: K-means was used as the clustering algorithm, the subsampling number was set to 50, k value varied between 2 and 9, and the proportion of each resampling was 80% of the total sample. Subsequently, we used the elbow method to determine the optimal k value of partition by evaluating the consensus matrix and the consensus cumulative distribution function. Within this analysis we were able to determine a suitable number of patients per cluster.

### Survival and enrichment analyses of patients stratified by lactate-related genes

LUAD patients were stratified in two different subtypes according to their expression of lactate-related genes. We used the survminer package of the R software to perform the survival analysis on the two identified subtypes. Kaplan-Meier (K-M) survival curves were employed to compare the survival of LUAD patients.

Hallmark gene sets (downloaded from the MSigDB database: http://www.gsea-msigdb.org) are rich in gene signatures of abundant biological states and processes. To investigate the biological activity and process patterns of LUAD samples, we performed a single sample gene set enrichment analysis (ssGSEA) of the two subtypes based on the hallmark gene set. The clusterProfiler package of R software (Yu et al., 2012) was used to calculate the enrichment scores.

### Tumor purity and immune infiltration of different subtypes

ESTIMATE is an algorithm that uses gene expression signatures to quantify the proportion of stromal and immune cells in tumor tissues. By using the estimate package of R software (Yoshihara et al., 2013), we obtained the immune score, stromal score, ESTIMATE score (the sum of immune and stromal scores), and tumor purity for each TCGA-LUAD sample in the two lactate-related subtypes.

To obtain the landscape of immune cell infiltration in LUAD tissues, we performed an ssGSEA on the expression matrix of TCGA-LUAD patients. By using the GSVA package of R software (Hanzelmann et al., 2013), we calculated the enrichment fraction of 28 immune cells in each sample.

### Construction of the lactate-related prognostic index

We identified genes with a differential expression among the two lactate-related subtypes by using the limma package of R software. Differentially expressed genes (DEGs) were identified as those genes presenting a logFC >2 and *p* < 0.05. Using the glmnet package in R software, we performed a least absolute shrinkage and selection operator (LASSO) analysis on the expression of DEGs in the train group (TCGA-LUAD cohort) to identify genes that could predict the overall survival of LUAD patients and establish a lactate-related prognostic index (LRPI). Then we used multivariate cox regression analysis for genes included in the model to verify their association with prognosis. Using the GEO cohort as the test group, we divided the train and test groups into two subgroups, namely an LRPI-high and an LRPI-low subgroup, using the median of the risk score as a cutoff. To verify the predictive ability of the model, we used the K-M method to perform a survival analysis of the two subgroups, used the timeROC package of R software to draw receiver operating characteristic (ROC) curves for the two subgroups at 1-, 2- and 3-year and calculated their area under the curve (AUC).

### Prognostic ability and stratified survival analysis of lactate-related prognostic index

To verify the independent prognostic ability of LRPI, we performed univariate and multivariate Cox regressions using the LRPI score and common clinical features in the TCGA-LUAD cohort. To improve the prognosis prediction of LUAD patients, we drew a nomogram using the rms package of R software. Finally, to obtain different survival characteristics, we stratified patients according to age, sex, and tumor stage and performed a K-M survival analysis.

### Molecular characteristics of the two identified LUAD subgroups

To obtain the mutation landscape of LRPI genes in both subgroups, we analyzed the simple nucleotide variation dataset within the TCGA-LUAD cohort using the Maftools package of R software (Mayakonda et al., 2018). We calculated the tumor mutation burden (TMB) for each sample and compared it between the two subgroups. To identify mutated genes that have a direct effect on tumor progression (driver mutated genes), we used the MutSigCV software of matlab (Lawrence et al., 2013). Subsequently, we performed a correlation analysis on the amount of mutations present in driver mutant genes, and calculated the correlation of these mutations.

### Drug sensitivity and immune therapy response

The Genomics of Drug Sensitivity in Cancer (GDSC) database (www.cancerrxgene.org/) was employed to assess the sensitivity of samples to different drugs by identifying biomarkers of drug sensitivity to different anti-cancer drugs.

Using GDSC, we predicted the IC50 of commonly used drugs in the treatment of LUAD patients. To assess the response of the two subgroups of patients to immune checkpoint therapy, we performed the Tumor Immune Dysfunction and Exclusion (TIDE) analysis to analyze the resistance to immunotherapy (Jiang et al., 2018).

### Statistical analysis

R software (version 4.1.1) (http://www.r-project.org/) and its corresponding R packages were used for all statistical data analysis and to generate graphs. A log-rank test was used to compare K-M survival curves for survival analysis. The Wilcoxon test was used to compare gene expression, ssGSEA analysis scores, ESTIMATE analysis scores, drug sensitivity, and TIDE analysis scores between two groups of samples. The Cox regression model was used to identify associated factors of survival outcomes. *p* values less than 0.05 were considered statistically significant.

## Results

### Stratification of LUAD patients according to their expression of lactate-related genes

**FIGURE 2:**
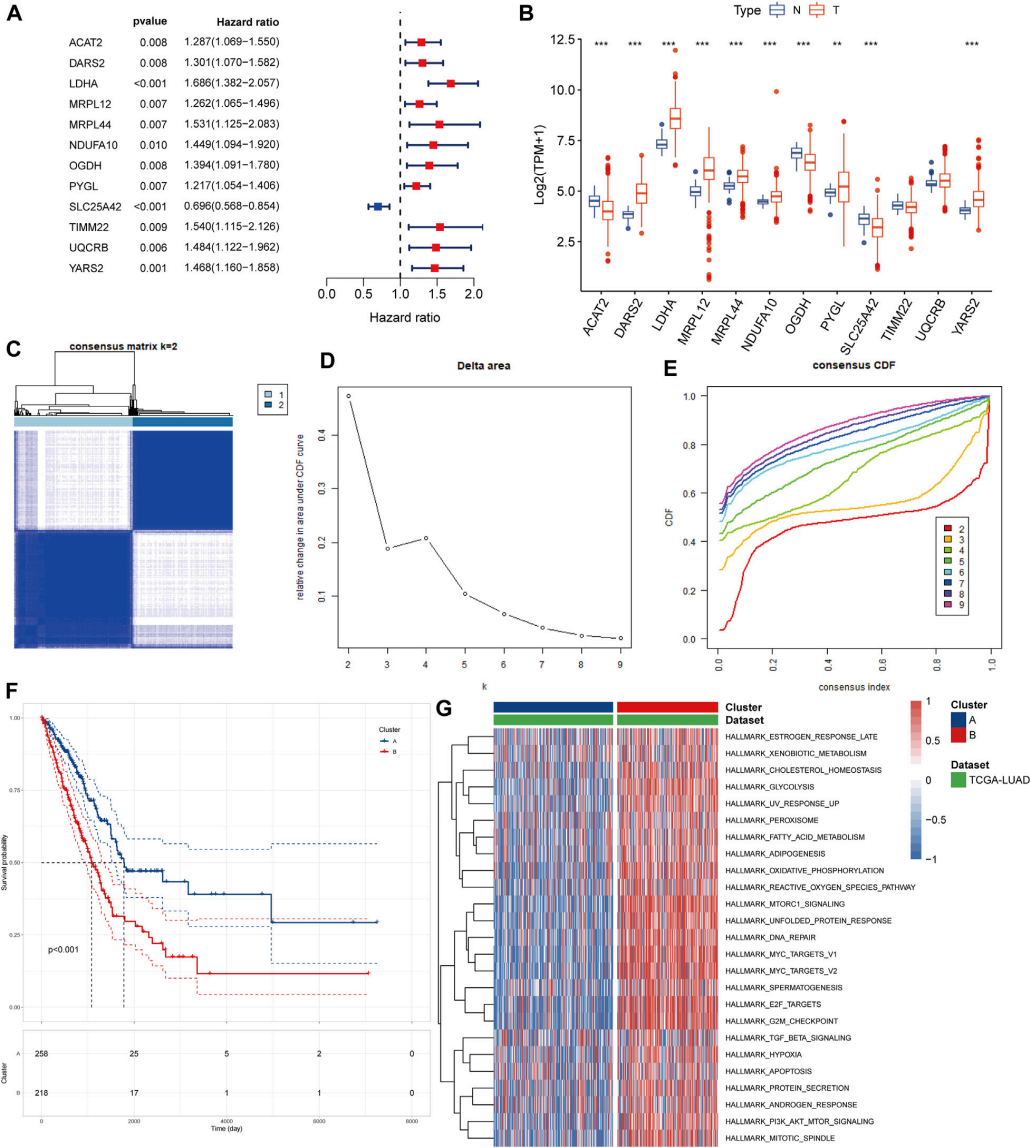
Identification of lactate-related subtypes of LUAD **(A)** Screening of prognostic lactate-related genes by univariate cox analysis **(B)** Comparison of expression values of lactate-related prognostic genes between normal samples and tumor samples **(C)** Consensus matrix heatmap when k = 2 **(D)** Delta area plot showed the relative change in area under CDF curve as the value of k changes **(E)** CDF plot showed the cumulative distribution function for different values of k **(F)** K-M survival curves showed that LSA patients have better prognosis than LSB patients **(G)** Different ssGSEA analysis results of two isoforms on hallmark gene sets, different colors represent the different ssGSEA scores.

In the univariate Cox regression of lactate-related genes we found a significant correlation between 12 genes and patient prognosis (Figure 2A). Among these genes, seven were upregulated and three were downregulated (Figure 2B). To identify different lactate-related subtypes of LUAD patients, we performed a consensus clustering of the TCGA-LUAD cohort based on the expression of these 12 identified genes (Figure 2C-E). Based on the optimal number of clusters k = 2, we divided patients into two subtypes: lactate-related subtype A (LSA, n = 258) and lactate-related subtype B (LSB, n = 218).

### Characteristics of the two identified subtypes of LUAD patients

**FIGURE 3:**
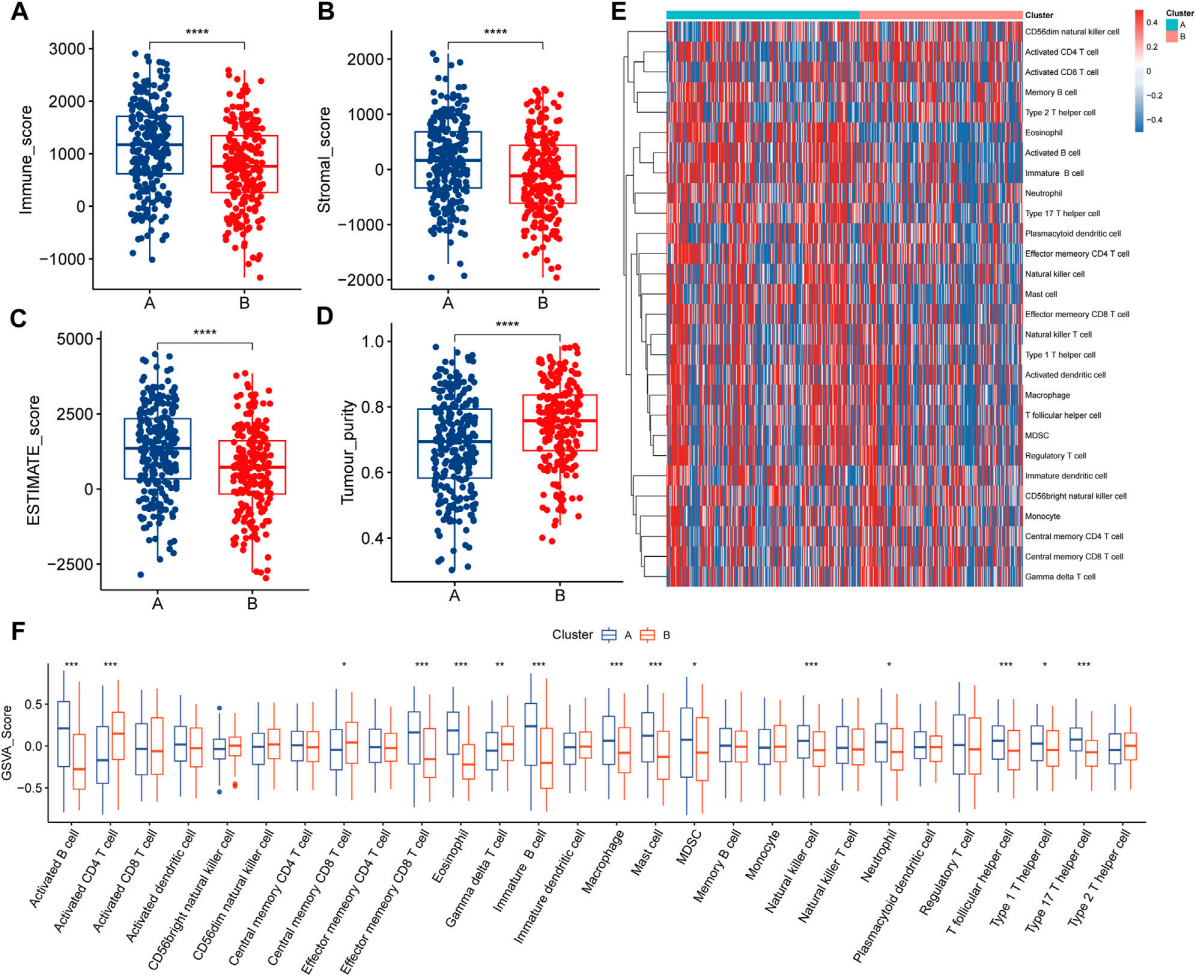
Immune characteristics of two lactate-related subtypes **(A–D)** Differences in the results of the four ESTIMATE analysis scores between the two subtypes **(E)** Landscape of 28 types of immune cell infiltration in patients with two subtypes, different colors represent different immune infiltration fractions **(F)** Differences in immune cell infiltration fractions in patients of two subtypes.

In the survival analysis, LSA patients showed better prognostic outcomes than LSB patients (Figure 2F). In parallel, LSB patients had a higher enrichment of lactate metabolism-related pathways, cell cycle-related pathways, PI3K/AKT/mTOR signaling pathways, and multiple cancer-related pathways in the ssGSEA analysis (Figure 2G); all of which may be associated with poor prognosis. Stromal and immune cells constitute an important part of the tumor tissue as they interact with tumor cells, and play an important role in tumor development and infiltration. Therefore, we explored differences in the TME between the two subtypes. Results of the ESTIMATE analysis showed that the immune, stromal, and ESTIMATE scores were higher in LSA samples, indicating that these samples displayed abundant immune cells and intercellular substance. Meanwhile, the tumor purity of LSB samples was significantly higher than that of LSA, indicating a higher proportion of tumor cells (Figures 3A– D). Immune cell infiltration analyses were in good agreement with ESTIMATE (Figure 3E). The overall level of immune cell infiltration in LSA was much higher than that of LSB samples. We found 12 cells that were more present in LSA samples and three cells that were more prevalent in LSB samples(Figure 3F). This suggests that, compared with LSB, LSA samples had more immune cells infiltrated, increased levels of interstitial components, and an active immune microenvironment that could significantly improve patient prognosis.

### Construction and validation of LRPI

**FIGURE 4:**
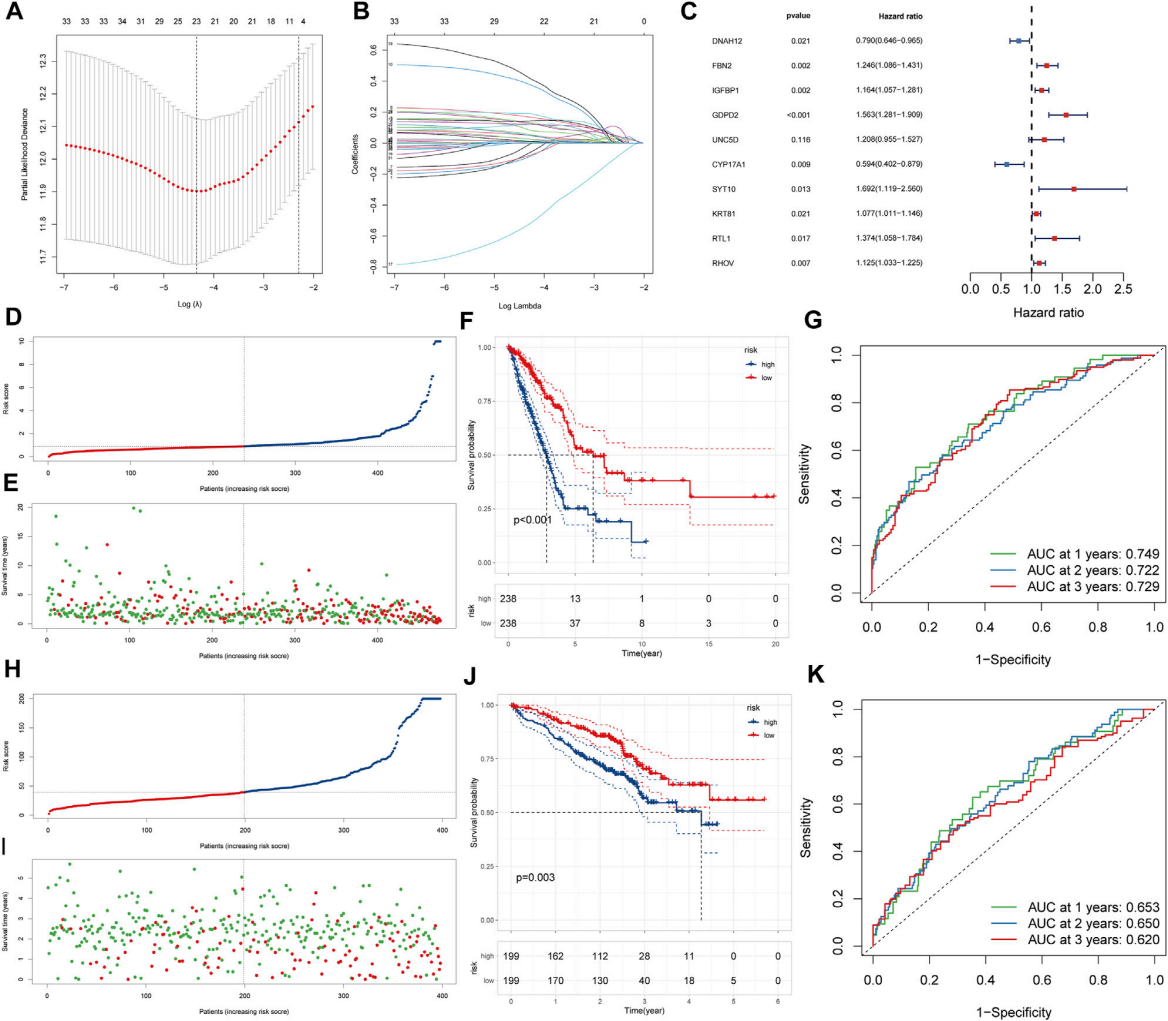
Construction and validation of LRPI **(A)** The change trajectory of the independent variable coefficient as the log lambda value increases **(B)** Change in misclassification probability as log lambda value changes **(C)**Multivariate cox analysis to validate the independent prognostic ability of model genes **(D and E)** LRPI score and survival outcomes of patients in the TCGA cohort **(F)** K-M survival curves of two LRPI subgroups of the TCGA cohort **(G)** 1-year, 2-years, and 3-years ROC curves and their area under the curve of LRPI in TCGA cohort **(H and I)** LRPI score and survival outcomes of patients in the GEO cohort **(J)** K-M survival curves of two LRPI subgroups of the TCGA cohort **(K)** 1-year, 2-years, and 3-years ROC curves and their area under the curve of LRPI in TCGA cohort.

To explore how gene expression affected the prognosis of patients in the two lactate-related subtypes, and to quantify differences in survival, we constructed an LRPI. First, the differential expression analysis of expression profiles of LSA and LSB patients identified 557 DEGs, which were further screened by the LASSO analysis (Figures 4A,B). Then, through multivariate Cox regressions, we found that genes DNAH12, FBN2, IGFBP1, GDPD2, UNC5D, CYP17A1, SYT10, KRT81, RTL1 and RHOV were significantly associated with prognosis (Figure 4C). Therefore, the LRPI (a hazard ratio regression model) was established based on these genes. The formula of the model is expressed as: LRPI = (−0.2361) * *expr*DNAH12 + (0.2201) * *expr*FBN2 + (0.1518) * *expr*IGFB1 + (0.4469) * *expr*GDPD2 + (0.1886) * *expr*UNC5D + (−0.5209) * *expr*CYP17A1 + (−0.5262) * *expr*SYT10 + (0.0738) * *expr*KRT81 + (0.3176) * *expr*RTL1 + (0.1176) * *expr*RHOV. Samples from the TCGA and the GEO cohorts were divided into two subgroups, namely LRPI-high and LRPI-low groups, based on their median model score (Figures 4D,E,H,I). The LRPI-low subgroup displayed a better prognosis than LRPI-high patients in both cohorts (Figures 4F,J). The AUC of the ROC curves at 1-, 3-, and 5-year in the TCGA cohort were respectively 0.749, 0.722, and 0.729 (Figure 4G), while corresponding values in the GEO cohort were 0.653, 0.650, and 0.620 (Figure 4K). Altogether, these data support that LRPI is an excellent prognostic indicator that can accurately predict the prognosis of LUAD patients.

### Independent prognostic ability and survival characteristics of LRPI

**FIGURE 5:**
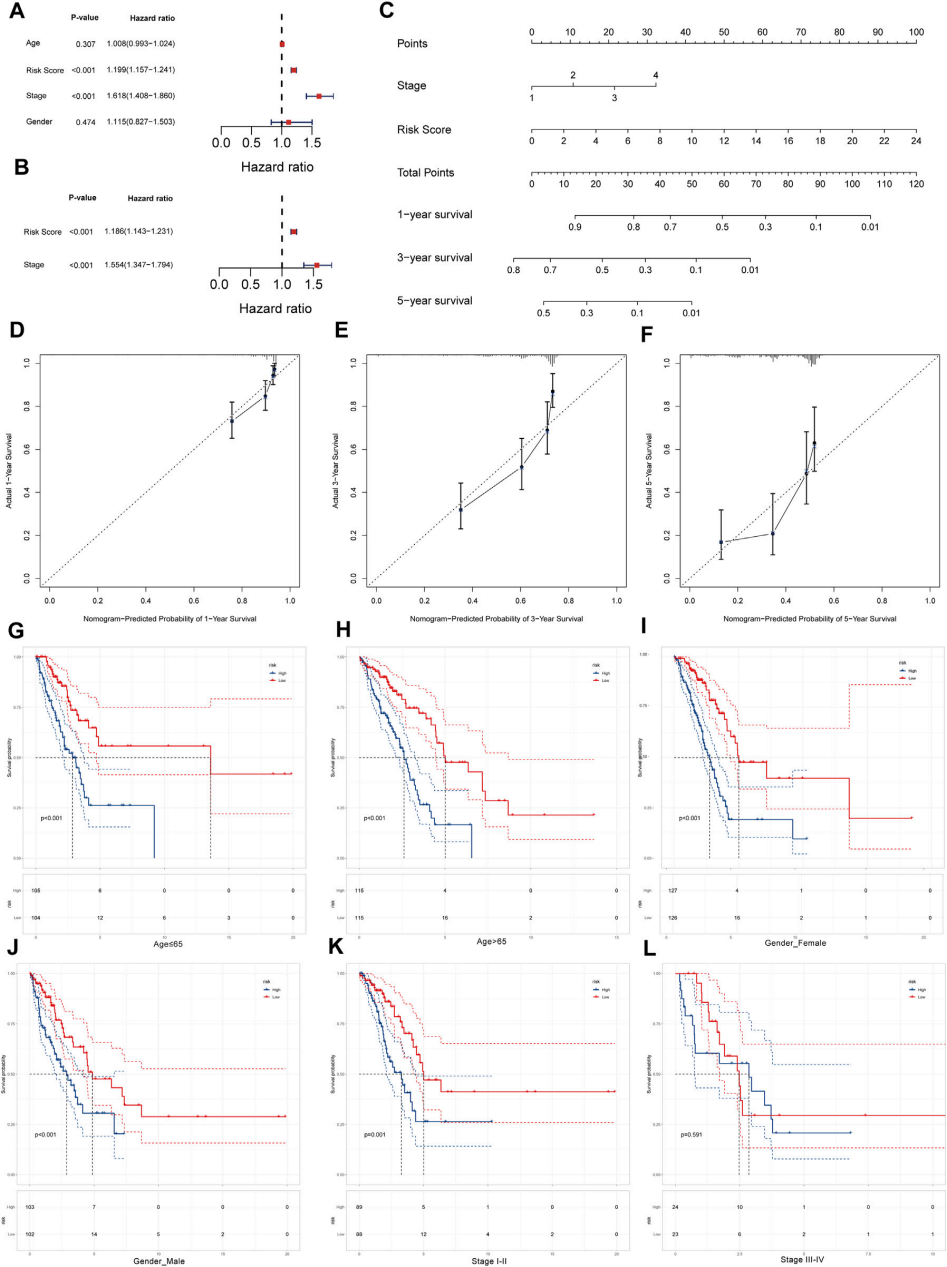
Survival prediction ability of LRPI and stratified survival analysis of two LRPI subgroups **(A and B)** Univariate and multivariate cox analysis to validate the independent prognostic power of LRPI **(C)** The nomogram was established by clinical stage and LRPI to predict the survival rate of patients at 1, 3, and 5 years **(D–F)** The calibration plot of nomogram at 1, 3, and 5 years **(G and H)** Age-stratified K-M survival curve analysis of two subgroups of LRPI, age was divided into two groups ≤65 and >65 **(I and J)** Gender-stratified K-M survival curve analysis of two subgroups of LRPI, gender is divided into female and male two groups **(K and L)** Stage-stratified K-M survival curve analysis of two subgroups of LRPI, clinical stage was divided into two groups, I-II and III-IV.

To test whether LRPI is an independent prognostic factor related to survival outcomes, we performed univariate and multivariate Cox regressions using age, LRPI, tumor stage, and gender (Figures 5A,B). Results evidenced that LRPI was an independent prognostic factor with a good prognostic ability. Subsequently, we established a prognostic nomogram for TCGA-LUAD patients. By adding points corresponding to the clinical stage and LRPI, the total score was used to predict the survival rate of patients at 1-, 3-, and 5-year (Figure 5C). Calibration plots for these time-points are shown in Figures 5D–F.

Next, we performed a stratified survival analysis to explore whether LRPI was an accurate predictor under different clinical factors. Survival of the LRPI-low subgroup was better than that of the LRPI-high subgroup in both cohorts of patients aged ≤65 years as well as in those aged ≥65 years (Figures 5G,H). The low LRPI subgroup also displayed an improved survival than the high LRPI subgroup in both cohorts when patients were stratified by sex (Figures 5I,J). When patients were stratified according to their clinical stage, we found that the survival of LRPI-low patients was better than that of LRPI-high ones for patients in stages I and II. However, there were no differences among subtypes in stage III or IV patients (Figures 5K,L). Altogether, results showed that LRPI could accurately predict the prognosis of patients stratified according to age and gender. With regards to clinical stage, LRPI was demonstrated to be an accurate predictor only for patients in stages I and II.

### Molecular characteristics of LRPI-high and LRPI-low subgroups

**FIGURE 6:**
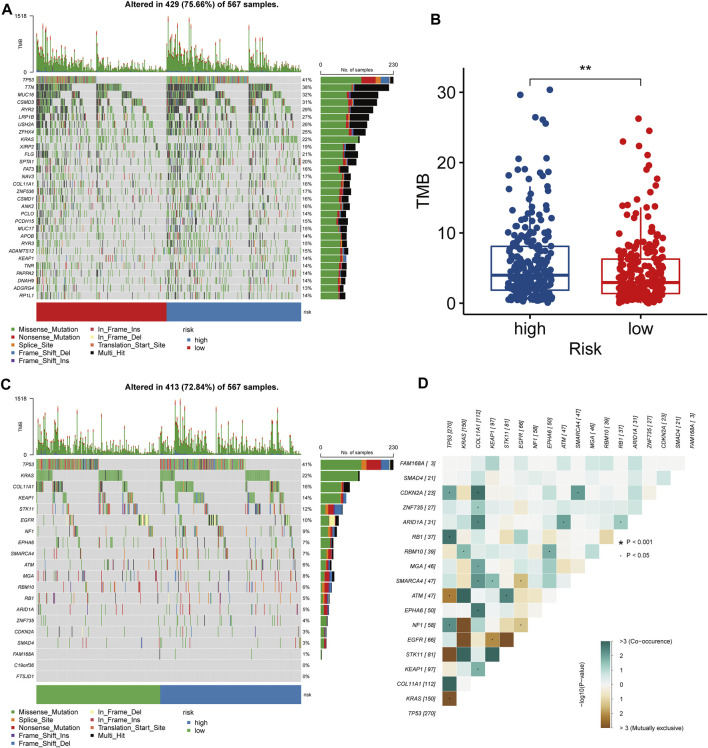
Molecular characteristics of two LRPI subgroups **(A)** The gene mutation landscape of two LRPI subgroups (different colors represent different mutation modes) **(B)** Boxplots showed the differences in TMB between two subgroups **(C)** Mutation landscape of driver-mutated genes of two LRPI subgroups **(D)** Correlations of mutation frequencies among driver-muted genes mutation.

We explored the overall mutational landscape of TCGA-LUAD and its distribution between the two subgroups according to their LRPI scores (Figure 6A). In the two subgroups, missense mutation, multi hit, and nonsense mutation occurred most frequently, while mutations of TP53, TTN, MUC16, and CSMD3 exceeded 30%. In this regard, TMB is an excellent biomarker to help predict the effect of immunotherapy. Interestingly, the LRPI-high subgroup showed a higher TMB (Figure 6B), suggesting that its response to immunotherapy was stronger than that of the LRPI-low subgroup. Next, we used MutSigCV to predict driver-mutated genes in LUAD patients (Figure 6C). The mutation frequency of TP53, KRAS, COL11A1, KEAP1, STK11, EGFR and other driver mutation genes was above 10%. Among driver mutant genes, COL11A1 showed a strong correlation with the mutations of CDKN2A, ZNF735, ARID1A, MGA, SMARCA4, EPHA6, KEAP1 and other genes. Therefore, COL11A1 might be a central gene that drives mutations in other genes in LUAD patients (Figure 6D).

### Treatment efficacy of LRPI-high and LRPI-low subgroups

**FIGURE 7:**
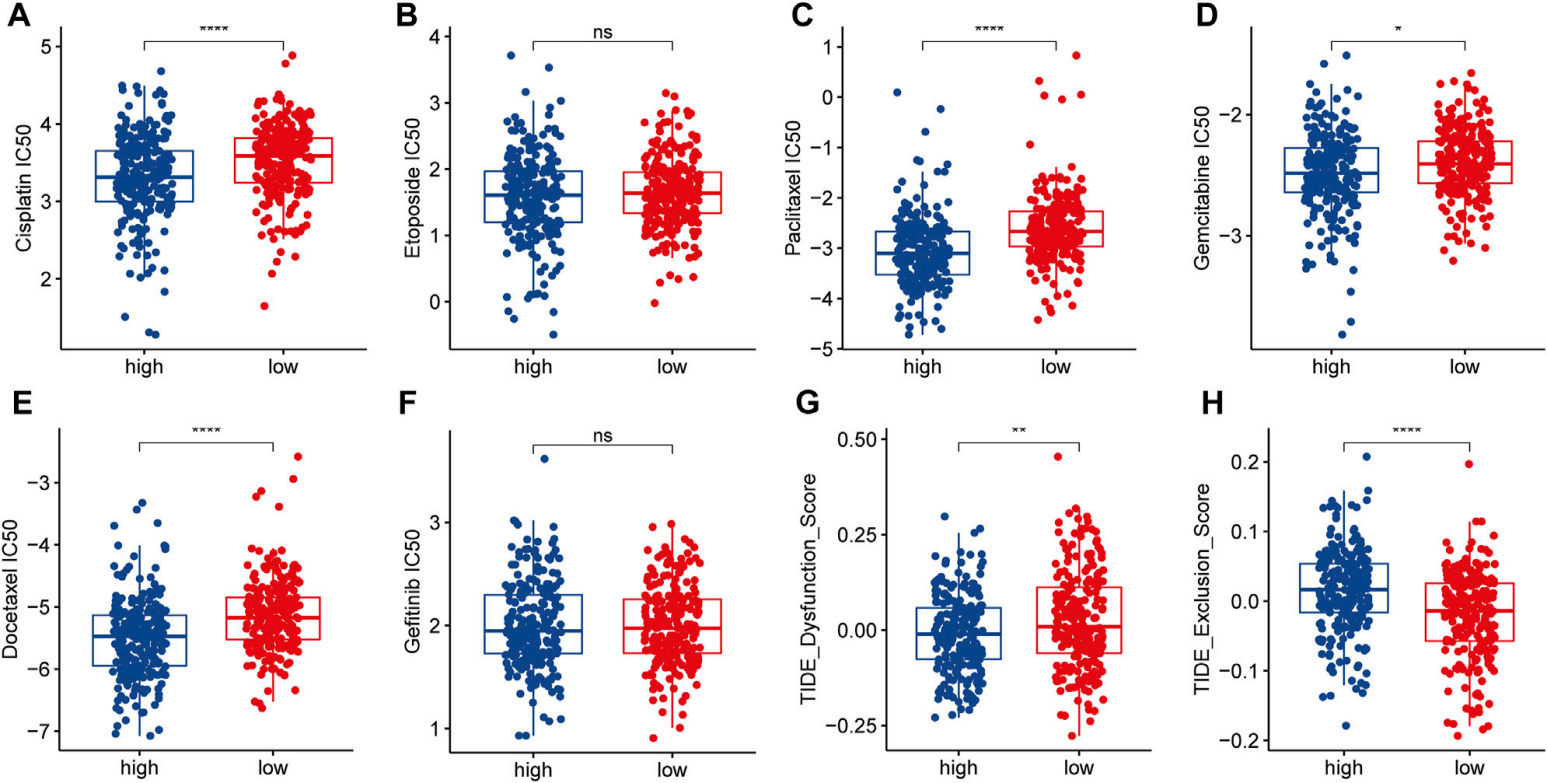
Response of two LRPI subgroups to antitumor therapies **(A–F)** Comparison of IC50 for different antitumor drugs (Cisplatin, Etoposide, Paclitaxel, Gemcitabine, Docetaxel, Gefitinib) in two LRPI subgroups **(G and H)** Comparison of dysfunction score and exclusion score in TIDE analysis of two LRPI subgroups.

In order to explore the sensitivity of the two identified patient subgroups to conventional treatment and to formulate improved treatment strategies, we performed a GDSC analysis to obtain the IC50 of different drugs (Figures 7A–F). Lower IC50 values mean better tumor responsiveness to anti-tumor drugs. Compared with the LRPI-low subgroup, the LRPI-high subgroup showed lower IC50 values for several chemotherapy drugs such as Cisplatin, paclitaxel, gemcitabine and docetaxel, indicating that these patients have an improved response to chemotherapy. Based on the results of drug sensitivity analysis, we recommend patients in LRPI-high subgroups to receive chemotherapy as adjuvant therapy. Compared with the LRPI-low subgroup, the LRPI-high subgroup displayed a lower TIDE dysfunction score (Figure 7G). Of note, the TIDE score has been correlated with lower responsiveness towards immunotherapy of hot tumors (Fu et al., 2020). Conversely, the LRPI-high group had a higher TIDE exclusion score (Figure 7H), suggesting a poorer response to immunotherapy in cold tumors (Fu et al., 2020).

## Discussion

LUAD is the leading cause of cancer-related deaths worldwide. Due to its high tumor heterogeneity, its carcinogenic mechanism has not been fully elucidated (Calvayrac et al., 2017; Huang et al., 2020), hindering efforts to develop individualized treatment. Recently, the role of lactate metabolism in multiple biological processes during tumor progression was revealed. Indeed, lactate metabolism is key to immune and inflammatory responses, leading to the development of tumor resistance to a variety of conventional, targeted, and immunological therapies (Balgi et al., 2011; Xie et al., 2016; Certo et al., 2021). Lactate was originally considered a metabolic waste product of glycolysis, until Otto Warburg first identified this metabolite as a characteristic product released by tumors. Considering the multiple roles of lactate metabolism in TME on tumor progression, and the impact of antitumor therapies, we believe it is essential to build a Biomarker based on lactate metabolism patterns in lung adenocarcinoma to predict patient survival outcomes, predict disease characteristics, and guide therapy. In our study, most of the lactate-related genes related to prognosis were upregulated in tumors when compared to normal tissues, which further confirmed the high correlation between lactate metabolism and tumor development. Therefore, the investigation of the effects of lactate metabolism on LUAD patients may uncover novel targets and biomarkers useful for individualized therapy. It would also be possible to generate models to predict patient survival and responsiveness to treatment and develop more efficacious treatment strategies.

In previous studies, lactate metabolism was highly correlated with tumor cell proliferation and invasion and patient poor prognosis (Faubert et al., 2017; Hui et al., 2017). In our study, LSB patients were enriched in lactate metabolism-related pathways and had a poorer prognosis than LSA patients. This is similar to results of a previous studies, confirming that increased lactate metabolism in LUAD is associated with poorer patient prognosis. In addition to promoting the development of tumor cells, lactate metabolism also influences the TME (Ippolito et al., 2019). Indeed, lactate negatively regulates the immune microenvironment, and displays a great inhibitory effect on the normal function of immune cells (such as the cytolysis function of T cells and NK cells) (Husain et al., 2013; Crane et al., 2014; Brand et al., 2016). Therefore, we analyzed immune infiltration within LSA and LSB LUAD patients. LSB patients, which displayed higher lactate metabolism, presented higher tumor purity, lower interstitial component and decreased immune cell components. In parallel, the majority of immune cells in the LSB subtype had a significantly lower infiltrating fraction, while only a few cells that do not play a major role in tumor immunity were enriched. These data validate the negative impact of lactate metabolism in LUAD as well as on the normal biological function of immune cells within TME.

Between the two LUAD subtypes, LSA and LSB, we found prognostic differences that were significantly associated with lactate metabolism. We next constructed models to predict patient outcome, explore molecular and immunological features and assess the efficacy of different treatment regimens correlated with lactate metabolism. Therefore, according to DEGs identified in the two subtypes, we screened model genes and established a prognostic model using LASSO and Cox regression analyses, so that the model (LRPI) could fully reflect differences according to lactate metabolism levels. LRPI was validated by the GEO cohort and was also shown to be an independent prognostic factor, prompting us to draw a nomogram based on two independent prognostic factors, LRPI score and clinical stage, to more accurately predict the survival rate of LUAD patients.

According to their LRPI score, LUAD patients were divided as LRPI-high and LRPI-low subgroups, which showed distinct prognoses. Interestingly, the prognosis of both subgroups was different when patients were stratified by age and gender, but were similar in stage III-IV patients. Indeed, higher clinical stage affects the prognosis of LUAD patients (Rami-Porta et al., 2018). In the multivariate Cox analysis, the hazard ratio of stage was greater than that of LRPI, indicating that higher clinical stages display collinearity with LRPI, thus masking its impact on prognosis.

Patients in the LRPI-high subgroup had improved sensitivity to a variety of drugs such as cisplatin, paclitaxel, gemcitabine and docetaxel, revealing that these patients would benefit more from chemotherapy than LRPI-low ones. The clinical use of immune checkpoint inhibitors has brought a new perspective to the treatment of lung cancers, and has shown excellent efficacy in NSCLC (Sharma and Allison, 2015), therefore we explored the relationship between the two subgroups and their benefit with regards to immunotherapy. The LRPI-high subgroup had a significantly higher TMB, which has been shown to be an important biomarker associated with a high sensitivity to immunotherapy (Samstein et al., 2019). Meanwhile, the LRPI-high subgroup presented a lower TIDE dysfunction score. Compared with the severe T cell dysfunction present in LRPI-low patients, LRPI-high patients showed improved sensitivity to immunotherapy. Based on these results, we recommend chemotherapy and immunotherapy for LRPI-high patients to improve their prognosis.

Although we extensively analyzed the role and impact of lactate metabolism in LUAD, there are two major shortcomings in the research. First, the study was based on bioinformatics analysis and lacked validation of the basic experiments, which we will further explore in future studies. Secondly, considering the different technologies and platforms used between transcriptomic datasets and the huge batch effect between different datasets, we used only the TCGA-LUAD dataset for the major analysis, which is one of the limitations of our article.

In conclusion, we established two LUAD patient subtypes with different levels of lactate metabolism, validating the role of lactate metabolism in the prognosis and immune function of LUAD, which is similar to that of other tumors. Based on differences in gene levels, we established a prognostic model to assess patient prognosis, molecular characteristics and response to treatment. LRPI could accurately predict the prognosis of LUAD patients, and, when combined to patient clinical stage, the accuracy of LRPI increased. Finally, LRPI can be used as a novel biomarker and as a tool for the individualized treatment of LUAD patients.

## Data Availability

The original contributions presented in the study are included in the article/supplementary material, further inquiries can be directed to the corresponding authors.

## Abbreviations

SCLC, small cell lung cancer; LUAD, Lung adenocarcinoma; TME, tumor microenvironment; Kaplan-Meier, K-M; ssGSEA, single ample gene set enrichment analysis; ESTIMATE, Estimation of stromal and immune cells in malignant tumor tissues using expression data; DEGs, differentially expressed genes; LRPI, lactate-related prognostic index; ROC, receiver operating characteristic; AUC, area under the curve; GDSC Genomics of Drug Sensitivity in Cancer; TIDE, Tumor Immune Dysfunction and Exclusion; ICB, immune checkpoint blockade.
